# The Hypertension Paradox: Survival Benefit After ST-Elevation Myocardial Infarction in Patients With History of Hypertension. A Prospective Cohort- and Risk-Analysis

**DOI:** 10.3389/fcvm.2022.785657

**Published:** 2022-02-24

**Authors:** Fabian Hoffmann, Patricia Fassbender, Wilhelm Zander, Lisa Ulbrich, Kathrin Kuhr, Christoph Adler, Marcel Halbach, Hannes Reuter

**Affiliations:** ^1^Department of Internal Medicine III, University of Cologne, Cologne, Germany; ^2^Department of Cardiovascular Aerospace Medicine, Institute of Aerospace Medicine, German Aerospace Center, Cologne, Germany; ^3^Department of Internal Medicine, Evangelisches Klinikum Köln Weyertal, Cologne, Germany; ^4^Medical Faculty, Institute of Medical Statistics and Computational Biology, University Hospital Cologne, Cologne, Germany; ^5^Fire Department City of Cologne, Institute for Security Science and Rescue Technology, Cologne, Germany

**Keywords:** STEMI (myocardial infarction), hypertension, mortality, RAS inhibitor, survival, beta blocker, prescription rate

## Abstract

**Background:**

Mortality after ST-elevation myocardial infarction (STEMI) is dependent from best-medical treatment after initial event.

**Objectives:**

Determining the impact of prescription of guideline-recommended therapy after STEMI in two cohorts, patients with and without history of arterial hypertension, on survival.

**Methods:**

1,025 patients of the Cologne Infarction Model registry with invasively adjudicated STEMI were dichotomized according to their history of arterial hypertension. We recorded prescription rates and dosing of RAS-inhibitors, β-blockers and statins in all patients. The primary outcome was all-cause death. Mean follow-up was 2.5 years.

**Results:**

Mean age was 64 ± 13 years, 246 (25%) were women. 749 (76%) patients had a history of hypertension. All-cause mortality was 24.2%, 30-day and 1-year mortality was 11.3% and 16.6%, respectively. History of hypertension correlated with lower mortality (hazard ratio [HR], @30 days: 0.41 [0.27-0.62], @1 year: 0.37 [0.26-0.53]). After adjusting for age, sex, Killip-class, diabetes mellitus, body-mass index, kidney function and statin prescription at discharge 1-year mortality HR was 0.24 (0.12-0.48). At discharge, prescription rates for RAS-inhibitors, β-blockers and statins, as well as individual dosing and long-term persistence of RAS-inhibitors were higher in patients with history of hypertension. On the same lines, prescription rates for RAS-inhibitors, β-blockers and statins at discharge correlated significantly with lower mortality regardless of history of hypertension.

**Conclusion:**

Patients with history of hypertension show higher penetration of guideline recommended drug therapy after STEMI, which may contribute to better survival. Better tolerance of β-blockers and RAS-inhibitors in patients with history of hypertension, not hypertension itself, likely explains these differences in prescription and dosing.

## Summary

### What Is Already Known on This Topic

Best medical treatment after ST-segment elevation myocardial infarction with statins, β-blockers and RAS-inhibitors contribute to better outcome concerning overall mortality and morbidity. A primary prophylactic potential of these drug is suspected.

### What This Study Adds

Higher prescription rates and dosages of guideline-recommended medication for secondary prevention show a major impact on survival after ST-elevation myocardial infarction.

The patient with history of arterial hypertension shows higher prescription rates and dosages of guideline-recommended secondary prophylactic medication after ST-elevation myocardial infarction and consecutively better survival.

Prescription of RAS inhibitors, β-blockers and statins seems to elicit a primary prophylactic potential of ST-elevation myocardial infarction.

## Introduction

The broader availability of cardiac catheterization laboratories, shorter transfer times for percutaneous coronary interventions and modern drug therapy with proven prognostic benefit in primary and secondary prevention are major achievements in the treatment of acute ST-elevation myocardial infarction (STEMI). Due to these measures the incidence of STEMI and the overall mortality due to ischemic heart disease has decreased in Europe and the United States in recent years (1, 2). Nevertheless, mortality remains high with a 1-year death rate of approximately 10% after STEMI (3, 4).

Arterial hypertension is known to be one of the major risk factors for the development of coronary artery disease and myocardial infarction. Inhibitors of the renin-angiotensin-system (RAS, either an ACE inhibitor or an AT1-receptor blocker), β-blockers, calcium channel blockers, and diuretics are recommended as the basis of antihypertensive treatment, since they all have demonstrated effective reduction not only of blood pressure but also cardiovascular events in randomized controlled trials (5). RAS inhibitors are particularly effective in reducing left ventricular hypertrophy and in ameliorating proteinuria, thus preserving renal function. Due to their high tolerance and wide availability also in single pill combinations, RAS inhibitors are widely used as first choice in antihypertensive treatment. On the other hand, β-blockers exhibit a somewhat less favorable side effect profile with a higher rate of treatment discontinuation compared to RAS inhibitors when assessed in real-life conditions (6). For the treatment of hypertension, β-blockers are therefore indicated primarily in clinical situations where they have shown to be particularly useful such as angina, post- myocardial infarction and heart failure (7, 8).

Early in the treatment of STEMI, current guidelines recommend a combination of dual antiplatelet therapy, a lipid-lowering regimen with statins, β-blockers and RAS inhibitors for secondary prevention (1, 2). For many patients, however, this poses a therapeutic dilemma. In a state of loss of cardiac function with a drop in cardiac output like myocardial infarction the effects of RAS inhibitors and β-blockers on blood pressure and heart rate may pose a serious risk (9). Hence, prescription rates as well as the dose prescribed are frequently reduced limiting the prognostic benefit of these agents for the individual.

We hypothesize that patients with a history of arterial hypertension at the time of hospital admission have better clinical condition and tolerance and therefore higher prescription rates and dosage for β-blockers and RAS inhibitors in secondary prevention following STEMI compared to normotensive patients. These differences in medication may directly influence short- and long-term outcome, and ultimately mortality.

## Materials and Methods

**Figure 1:**
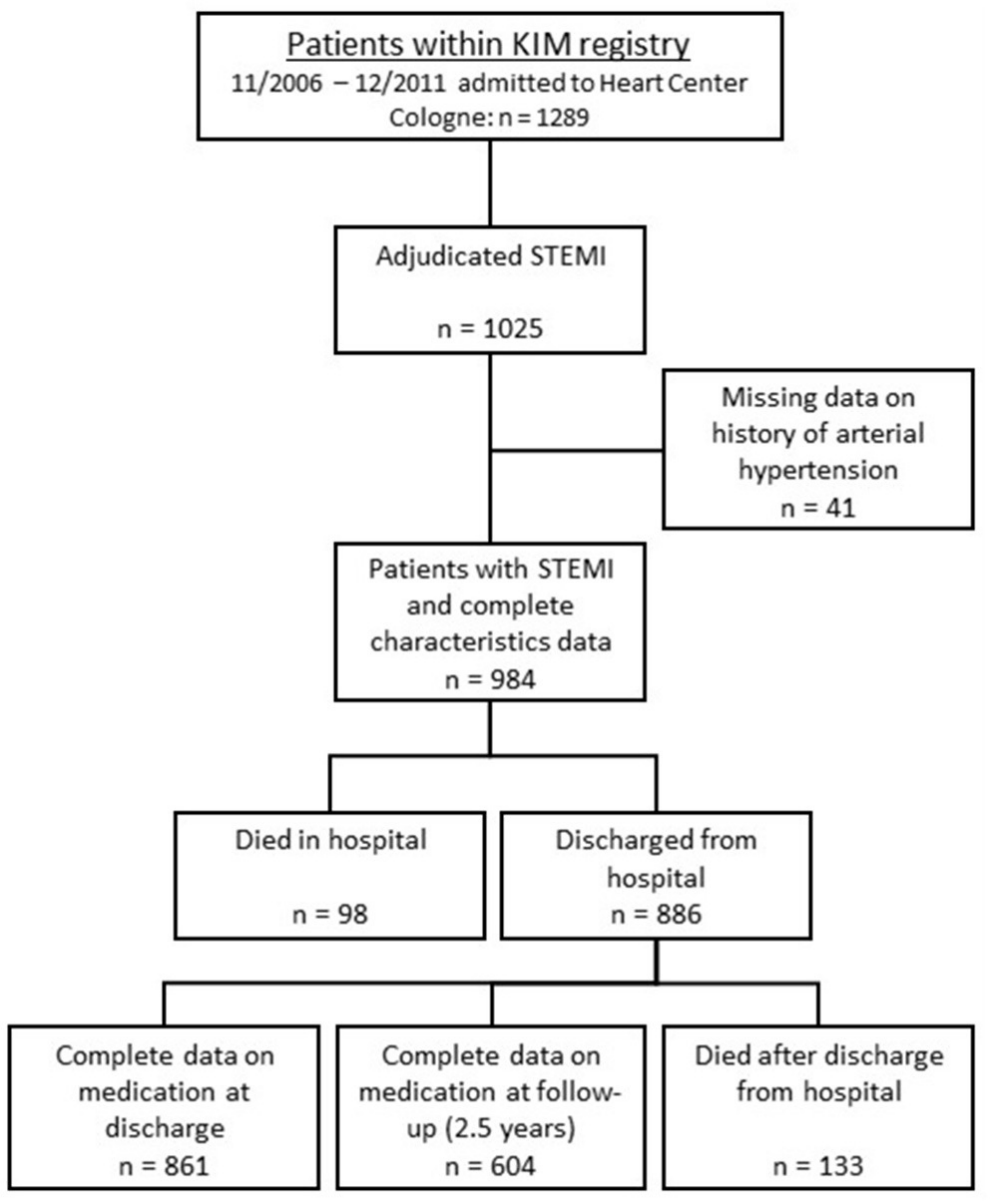
Scheme of group distribution within the KIM-registry. KIM—Cologne Infarction Model; STEMI—ST-elevation myocardial infarction.

This study is a prospective analysis of patients being referred to the Heart Center of Cologne as part of the treatment protocol of KIM (Cologne Infarction Model). KIM is a regional network of the 16 hospitals and the emergency medical services of the city of Cologne that aims at optimizing standard of care for patients with clinical and electrocardiographic signs of STEMI by shortening transfer times after symptom onset. According to protocol these patients identified by emergency medical services are directly transferred to one of five catheterization laboratories with 24-h service within city limits, one of which is the Heart Center of the University of Cologne. Transfer times and initial treatment are documented in standardized forms and entered into an electronic database. Details on the KIM registry have previously been described (10). Between November 2006 and December 2011, 1,289 consecutive KIM patients were admitted to the Heart Center of the University of Cologne and underwent acute coronary angiography. In 1,025 patients diagnosis of STEMI was hereby confirmed, providing a consecutive and homogenous cohort for the present study (Figure 1). Patients' data and characteristics were retrieved from the KIM database for the present analysis. The data set was completed by reviewing patients' hospital records. All data were stored anonymously. Between August 2012 and March 2013 follow-up data were acquired. By using a standardized questionnaire, current medication, changes in cardiovascular risk, further cardiovascular events or the need for reintervention during the follow-up period were enquired. These forms were either completed by the patient himself or information was acquired via phone interview. The interviews were performed by trained staff and in case that the patient could not be reached data were obtained by contacting the patients' suggested contacts or primary care physicians. For confirmation of death we consulted the health insurance database. Mean follow up was 2.5 ± 1.5 years.

Mortality endpoints were defined as all-cause mortality for the whole duration of the follow-up period, 30-day mortality and 1-year mortality starting at time of admission to the hospital in the acute event. Persistence was calculated for statins, β-blockers and RAS inhibitors individually. Persistence was defined as the fraction of patients with prescription of the same medication class at discharge and follow-up regardless of dosage. Dosages of medication were quantified at time of discharge and documented as defined daily dose; a description of the prescribed fraction of the maximum daily dose of the individual medication.

The present study complies with the Declaration of Helsinki 1975, revised Hong Kong 1989 and was approved by the local ethics committee (University of Cologne 06-064). Written informed consent was obtained from all patients.

### Statistical Analysis

Baseline characteristics are displayed as mean with their first standard deviation. We dichotomized our cohort according to a documented history of arterial hypertension at time of hospital admission. A survival analysis using hazard ratios and the corresponding 95%-confidence interval, logistic- and Cox- regression with a Kaplan-Meier-analysis was done for patients with history of arterial hypertension in comparison to patients without. We adjusted the mortality analysis model by including further established cardiovascular risk factors: age, sex, kidney function, prevalence of diabetes mellitus and body mass index. Furthermore, we included individual Killip-class and prescription rate of statins for their known impact on mortality after STEMI. To define subgroups at risk, according to baseline characteristics, we calculated relative risk with confidence interval. Comparison of continuous data was done using ANOVA. Dichotomous data were analyzed using X^2^-test. Level of significance was *p* < 0.05. Statistical analysis was performed using SPSS 23 (IBM, New York, USA).

## Results

One thousand and twenty five consecutive patients with STEMI were dichotomized according to their history of arterial hypertension at hospital admission. Mean age was 63.5 ± 13.2 years, 246 (24.5%) were female, 749 (76.1%) had a documented history of arterial hypertension. Table 1 displays the baseline characteristics of the whole and dichotomized cohorts. With significantly higher age, body mass index, higher incidence of diabetes mellitus and hyperlipoproteinaemia patients with a history of hypertension presented with an overall stronger individual cardiovascular risk compared to patients without. Forty one patients had to be excluded due to lack of data concerning their history of hypertension (Figure 1).

**Table 1:** Baseline characteristics.

**Parameter**	**Unit**	**Total** ***n* = 1,025**	**Hx of hypertension *n* = 749**	**No hx of hypertension** ***n* = 235**	***P*-value hx vs. no hx of hypertension**
Female Sex	[%]	24.5	25.8	20.0	0.072
Age	[years]	63.5 ± 13.2	64.6 ± 12.8	59.7 ± 13.8	<0.001
Body mass index	[kg/m^2^]	27.4 ± 5.1	27.6 ± 5.1	26.5 ± 5.1	0.023
Glomerular filtration rate	[ml/min]	86.6 ± 32.1	86.2 ± 32.5	87.6 ± 30.6	0.582
Diabetes mellitus	[%]	25.0	29.0	12,3	<0.001
Smoker^*^	[%]	21.1	19.3	31.5	0.012
Hyperlipoproteinaemia^*^	[%]	87.8	89.6	77.5	0.001
Anterior MI	[%]	48.8	47.3	53.6	0.092
Left-main Infarction	[%]	2.7	2.9	2.6	0.143
Prior heart failure	[%]	7.4	8.0	5.5	0.212
Prior bypass	[%]	4.9	6.2	1.7	0.090
LV-ejection fraction	[%]	60.8 ± 17.2	60.6 ± 17.6	61.7 ± 15.6	0.744
Max. creatine-kinase	[U/l]	2,004 ± 3,584	1,792 ± 3,020	2,707 ± 4,969	0.001
Coronary multivessel disease	[%]				
No		38.8	38.2	40.6	
Yes		61.1	61.8	59.4	
Overall					0.295
Killip-class	[%]				
1		79.9	82.9	68.5	
2		0.6	0.7	0.4	
3		1.5	1.3	2.1	
4		18.0	15.1	28.9	
Overall					<0.001
Number of acute interventions	[%]				
1		83.2	83.0	83.8	
2		7.8	8.3	6.4	
3		8.9	8.7	9.8	
Overall					0.583
Systolic blood pressure^∧^	[mmHg]	130.6 ± 30.4	133.7 ± 29.6	120.4 ± 30.9	<0.001
Diastolic blood pressure^∧^	[mmHg]	75.3 ± 18.1	75.9 ± 17.9	73.2 ± 18.5	0.043
Heart rate^∧^	[bpm]	82.4 ± 24.2	81.5 ± 23.7	85.2 ± 25.7	0.044

*Comparison of the whole cohort and subgroups dichotomized according to a diagnosed history of hypertension at time of admission (Hx of hypertension vs. no hx of hypertension)*.

*MI, myocardial infarction; LV, left ventricular. Plus–minus values are means ±SD*.

* *Assessed at follow up (mean 2.5 years)*.

∧ *Assessed at time of admission. Level of significance: p < 0.05*.

After a mean follow up of 2.5 years all-cause mortality was 24.2 %. Post STEMI, in-hospital, 30-day and 1-year mortality was 10, 11.3, and 16.6%, respectively. Beside the higher prevalence of arterial hypertension, survivors at all predefined time points were younger, had a better kidney function, had less severe coronary artery disease and lower Killip-class (Table 2). Additionally, 30-day and 1-year survival was higher in males and in patients with lower peak creatine kinase. Having undergone a bypass procedure prior to the event decreased the chance of 1-year survival.

**Table 2:** Survival characteristics.

		**Total mortality**			**30-day mortality**			**1-year mortality**		
**Parameter**	**Unit**	**Deceased**	**Survivors**	***P*-value**	**Deceased**	**Survivors**	***P*-value**	**Deceased**	**Survivors**	***P*-value**
Hypertension	[%]	61.5	81.2	<0.001	59.8	78.4	<0.001	59.5	79.8	<0.001
Age	[years]	72.1 ± 12.1	61.0 ± 12.4	<0.001	72.8 ± 12.2	62.5 ± 12.9	<0.001	71.1 ± 12.4	62.1 ± 12.9	<0.001
Female sex	[%]	35.2	21.3	<0.001	46.0	22.1	<0.001	40.5	21.2	<0.001
Body mass index	[kg/m^2^]	26.9 ± 6.9	27.6 ± 4.7	0.149	28.6 ± 8.5	27.5 ± 4.9	0.238	26.8 ± 7.9	27.6 ± 4.7	0.204
Glomerular filtration rate	[ml/min]	66.9 ± 30.2	92.2 ± 30.6	<0.001	61.9 26.9	89.0 31.6	<0.001	62.0 ± 26.9	90.9 ± 31.0	<0.001
Diabetes mellitus	[%]	26.4	25.0	0.741	22.4	25.9	0.516	25.8	25.4	0.925
Prior heart failure	[%]	10.4	6.6	0.077	12.1	6.9	0.085	9.8	7.1	0.226
Prior bypass	[%]	9.1	3.9	0.002	4.7	5.1	0.970	9.8	4.1	0.002
LV-ejection fraction	[%]	52.9 ± 19.5	62.7 ± 15.8	0.003	57.2 ± 15.4	61.2 ± 17.03	0.454	54.3 ± 17.8	61.7 ± 16.7	0.079
Maximum creatine-kinase	[U/l]	2,744 ± 5,813	1,793 ± 2,662	<0.001	4,007 ± 8,719	1,811 ± 2,603	<0.001	3,159 ± 6,893	1,802 ± 2,637	<0.001
Coronary mulitvessel disease	[%]									
No		25.7	43.0		24.3	40.7		22.3	42.1	
Yes		74.3	56.9		75.6	59.3		77.7	57.8	
overall				<0.001			<0.001			<0.001
Killip-class	[%]									
1		54.5	88.2		30.1	86.3		41.7	87.2	
2		0.4	0.7		0.0	0.7		0.6	0.6	
3		3.7	0.7		3.5	1.2		3.1	1.1	
4		41.4	10.4		66.4	11.8		54.6	11.1	
overall				<0.001			<0.001			<0.001

*Survival characteristics for different timepoints (total, 30-day and 1-year mortality). Comparison of deceased vs. survivors. LV, left ventricular. Level of significance: p < 0.05*.

### Dichotomization According to History of Arterial Hypertension

Upon hospital admission in the acute event, Killip-class differed significantly between groups. Strikingly, patients with history of hypertension had a lower Killip-class and lower peak values for creatine kinase as surrogate for infarction size, on average. Ejection fraction, history of heart failure, prior coronary artery bypass graft procedures and quantitative assessment of coronary artery disease did not differ between groups. At time of admission, patients with history of hypertension presented with higher systolic (Δ 13.3 mmHg) and diastolic (Δ 2.7 mmHg) blood pressures, whereas heart rate was lower compared to patients without history of hypertension (Δ −3.7 bpm, all *p* < 0.05, Table 1).

**Figure 2:**
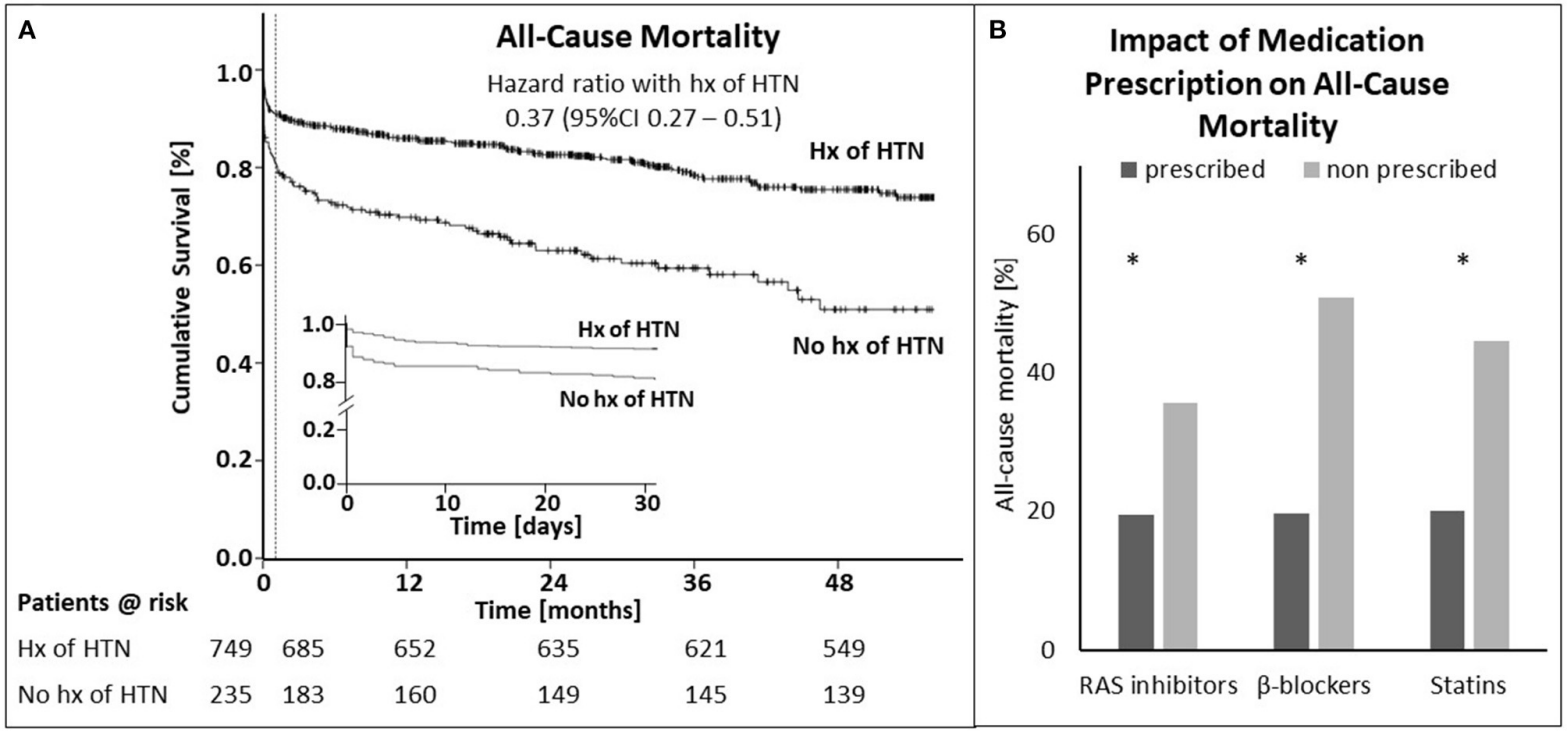
History of hypertension elicits survival benefit after STEMI. **(A)** Shown is the cumulative survival for patients with and without history (hx) of hypertension (HTN) from admission to end of follow-up **(A)** and during the first 30 days [**(A)**, inset]. The dotted line in **(A)** marks the 30-day time point. **(B)** Displays the impact of prescription of RAS inhibitors, β-blockers and statins for the whole cohort, except for patients who died within 24 h after admission to the hospital on mortality for. Asterisks indicate significance (*p* < 0.05). STEMI—ST-elevation myocardial infarction.

History of arterial hypertension at hospital admission significantly correlated with overall survival (HR for mortality 0.37, 95% confidence interval [CI] 0.27-0.51, *p* < 0.001, Figure 2A). The same could be observed for 30-day (0.41, 95% CI 0.27 – 0.62, *p* < 0.001) and 1-year mortality (0.37, 95% CI 0.26 – 0.53, *p* < 0.001; Supplementary Table 1 in Appendix). Even after adjusting for age, sex, diabetes mellitus, Killip-class, kidney function, body mass index and prescription rate of statins upon discharge, patients with history of arterial hypertension still had a significant survival benefit within 30 days from hospital admission (0.28, 95% CI 0.09 – 0.85, *p* = 0.025) and after 1 year (0.24, 95% CI 0.12 – 0.48, *p* < 0.001; Supplementary Table 1 in Appendix). When analyzing the individual parameters the logistic regression model for 30-day mortality revealed a significant impact of Killip-class, age and sex besides the history of hypertension. Cox-regression analysis of 1-year mortality showed significant impact of the above mentioned as well as kidney function and body mass index.

### Subgroup Analysis

**Figure 3:**
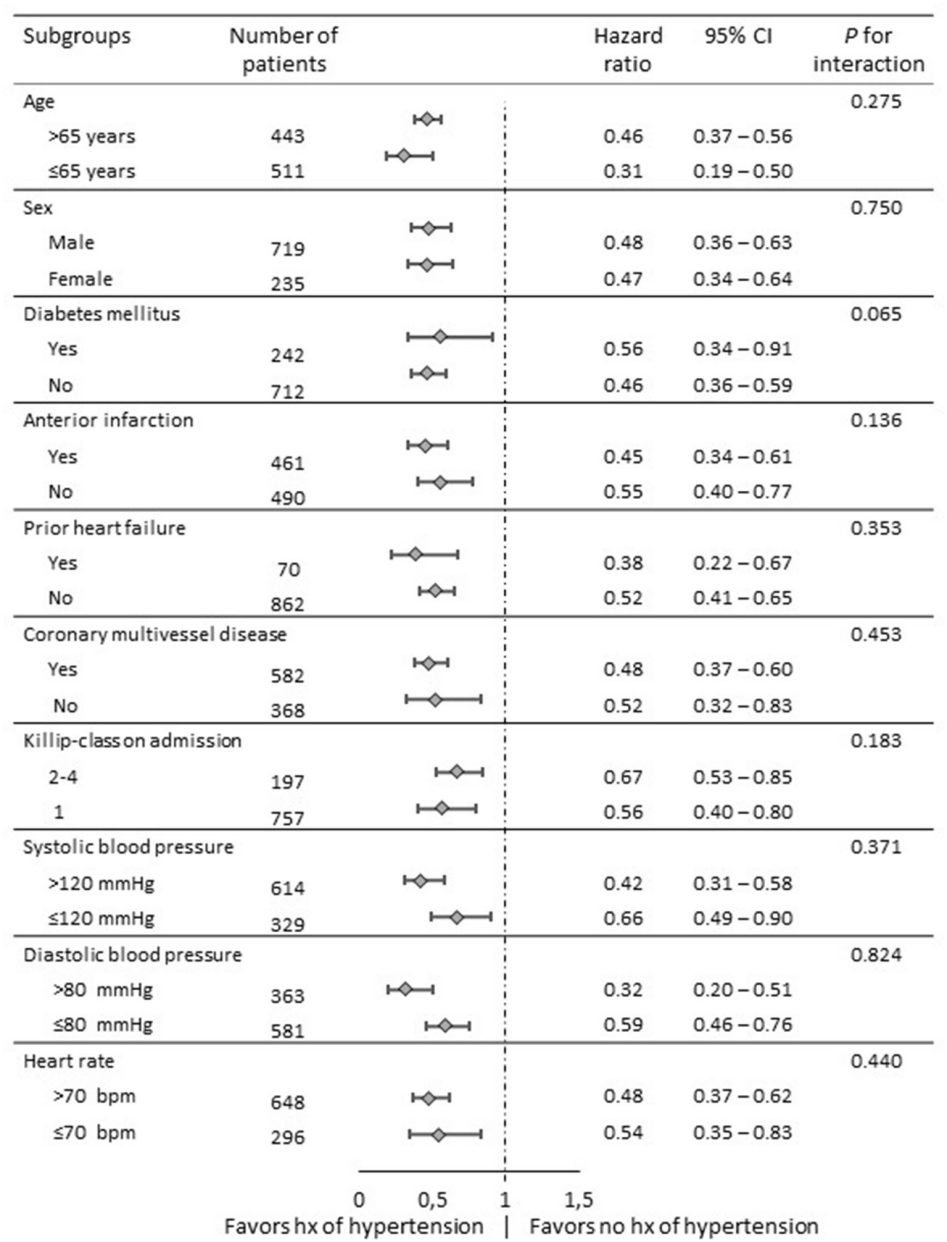
Comparative hazard ratios of all-cause mortality for subgroups in overall populations. Level of significance: *p* < 0.05. CI: confidence interval.

To determine whether the outcomes according to hypertension history observed in the overall population were consistent, we calculated the adjusted HR for death in various complex subgroups (Figure 3). The association with better outcome was consistent across these subgroups including patients with anterior myocardial infarction and high-risk populations such as patients with Killip-class ≥2, diabetes mellitus or multivessel disease. Of note, patients with history of hypertension showed better survival even in the subgroup of patients with blood pressures below 120mmHg systolic or 80mmHg diastolic at time of presentation.

### Medication for Secondary Prevention

Post STEMI at discharge all patients received dual antiplatelet therapy or a combination of oral anticoagulant plus mono or dual antiplatelet therapy per protocol (Supplementary Table 2 in Appendix). As potential explanation for the observed survival benefit in hypertensive patients we analyzed impact of RAS inhibitors, β-blockers and statins on mortality in the whole cohort. Additionally, we calculated prescription rate, persistence and dosing of these drugs for patients with and without history of hypertension. Figure 2B displays the impact of prescription of RAS inhibitors, β-blockers and statins on total mortality of the whole cohort, except for patients who died within 24 h after admission to the hospital. Mortality was lower for all medication groups when being prescribed at discharge (RAS inhibitors: 19.6 vs. 35.7%; β-blockers: 19.8 vs. 50.8%; statins: 20.1 vs. 44.6%). These differences were significant for all three comparisons (*p* < 0.001).

**Figure 4:**
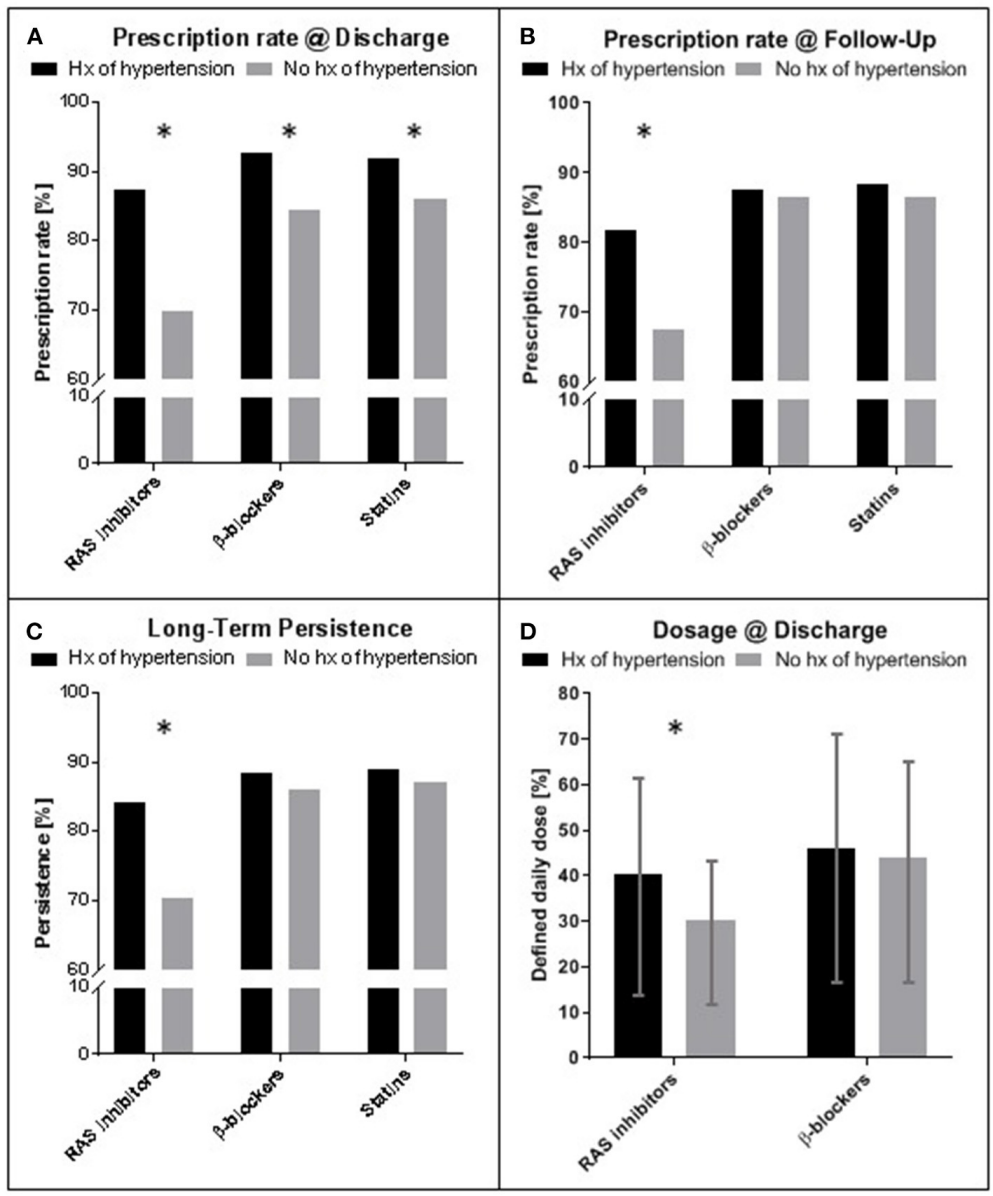
Prescription rate, persistence and dosing of medication for secondary prevention after STEMI. **(A)** Prescription rates at time of discharge for patients with and without history (hx) of arterial hypertension. **(B)** Prescription rates at time of last follow up (mean 2.5 years). **(C)** Long-term persistence measured as percentage of patients with prescription at discharge and follow up of the individual medication. **(D)** Comparison of dosing for RAS inhibitors and β-blockers at time of discharge. Hx of hypertension: history of hypertension; RAS, Renin Angiotensin system; Mean ± standard deviation. Asterisks indicate significance (*p* < 0.05). STEMI—ST-elevation myocardial infarction.

As shown in Figure 4, patients with history of hypertension had significantly higher prescription rates for all three medication groups at discharge (β-blockers: 92.8 vs. 84.3%, *p* < 0.001; RAS inhibitors: 87.3 vs. 69.8% *p* < 0.001; Statins: 91.9 vs. 85.9% *p* = 0.010, Figure 4A). Upon a mean follow up of 2.5 years rates for β-blockers and statins did not differ between groups, whereas RAS inhibitor rates were still higher in patients with history of hypertension (81.7 vs. 67.4%, *p* = 0.003, Figure 4B). Persistence to medication in these patients was significantly higher for RAS inhibitors (84.1 vs. 70.3%, *p* = 0.007, Figure 4C), but not for β-blockers or statins, accordingly. Average dosing for RAS inhibitors, but not for β-blockers at time of discharge was higher in patients with a history of hypertension (RAS inhibitors, defined daily dose: 40.2 ± 23.5% vs. 30.3 ± 15.4%, *p* < 0.001, Figure 4D).

## Discussion

Our findings show that even in a cohort of STEMI patients triaged for a primary percutaneous coronary intervention strategy with minimal system delay the prescription rate of medication for secondary prevention at discharge correlates with short- and long-term prognosis. We hypothesize that patients with arterial hypertension have a better tolerance and clinical condition allowing administration of β-blockers and RAS inhibitors earlier and at higher doses after the index event, likely conveying the survival benefit that we found in patients with a history of hypertension. This is even more remarkable since patients with a history of hypertension had a higher prevalence of metabolic syndrome, translating into an overall higher cardiovascular risk.

The strengths of this study are the prospective trial design enrolling a large and homogeneous group of STEMI patients in a metropolitan area with high standard of care based on a regional network designed to deliver 24/7 percutaneous coronary intervention-mediated reperfusion therapy. Furthermore, at time of discharge all patients were on dual antiplatelet therapy and had a high prescription rate for further guideline-recommended drug therapy.

With the high standard of care and the strong long-term adherence to recommended drugs in our STEMI registry, a 1-year mortality of 16.6% seems relatively high. This may be explained by the increased individual cardiovascular risk compared to previously published cohorts (3).

## Risk Factors and Preventive Medication

With an overall prevalence of 30–45% (5) arterial hypertension is one of the most common contributors to individual cardiovascular risk. Most often hypertension co-exists with augmenting factors such as hyperlipoproteinaemia, overweight or diabetes mellitus. This matrix of risk factors is a major contributor to acute cardiovascular events including myocardial infarction and stroke and shows a continuous relationship between blood pressure and event rate (5). Not to our surprise, the prevalence of hypertension is especially high in patients with STEMI regardless of age and sex (11). Hence, blood pressure control, especially with RAS inhibitors, β-blockers, calcium channel blockers and diuretics, is a cornerstone in primary and secondary prevention of cardiovascular events (5). The same has unequivocally been demonstrated for lipid-lowering therapy with statins, showing reductions in cardiovascular morbidity and mortality. In primary prevention, indication and dosing of statins should be tailored according to the individual risk. Post STEMI, it is recommended to start high-intensity statin therapy in patients as early as possible and maintain it long-term as secondary prophylaxis (1). In our cohort patients with a history of arterial hypertension had a higher prescription rate for statins at discharge (Figure 4A). This group also presented a higher cardiovascular risk overall. Hence, these patients more likely were under statin therapy already at time of presentation and may therefore have shown better tolerance toward this class of drugs as well as being in a better clinical condition upon admission. As shown by multivariable analysis, differences in prescription rates for statins did not alter the effect of hypertension status at time of admission on mortality outcomes in the present study.

## Role of Early vs. Delayed Treatment on Outcome

RAS inhibitors and β-blockers provide a prognostic benefit in patients with coronary artery disease or heart failure and are therefore recommended as the antihypertensive agents of choice in patients with cardiovascular comorbidities (5). Figure 2A clearly displays a survival benefit in hypertensive patients already on the day of the event. In acute myocardial infarction even pre-event use of RAS inhibitors and β-blockers may confer cardioprotective effects. An already established therapy with RAS inhibitors reduced myocardial infarct size determined by maximal troponin levels in patients with STEMI (12) and non-STEMI (13) independent of blood pressure control. Furthermore, pretreatment with RAS inhibitors has been shown to attenuate ischemia-reperfusion injury during coronary revascularisation (14). The benefit of RAS inhibition on mortality and the onset of heart failure, on the other hand, is likely attributed to reduced post-infarct remodeling of the left ventricle and appears to be more evident on long-term follow-up (15, 16). The timing of treatment initiation in this respect is equally important. In a meta-analysis by Rodrigues et al., trials of RAS inhibition initiated within 48 hours of myocardial infarction showed a significant mortality reduction by 7% at 30-day and an even larger reduction in 1-year mortality, however, this effect was attenuated in trials with RAS inhibition started more than 48 H after infarction (Odds ratio for 1-year mortality: 0.68 vs. 0.84) (15).

β-blockers, on the other hand, confer immediate benefit by blunting sympathetic activation after myocardial infarction in response to anxiety, pain and reduced contractility. This acutely reduces myocardial oxygen demand, limits infarct size, increases the threshold for malignant arrhythmia, and prevents maladaptive remodeling including heart failure on the long term (17, 18). Reperfusion itself is extremely effective in reducing sympathetic activity acutely (19). Nevertheless, contemporary studies confirm the additional benefit of early β-blockade. In a large prospective registry on STEMI patients treated with primary percutaneous coronary intervention, β-blocker therapy at discharge was associated with a 1.5% absolute reduction in all-cause death (20). Bugiardini and coworkers provided further evidence that this benefit is time sensitive. In a total of 5.259 patients with acute coronary syndrome, 71% of whom presented with STEMI, the probability of improving Left ventricular function and in-hospital survival was significantly higher if oral β-blockers had been administered within 24 h from admission compared to delayed treatment (>24 h) (21).

Although we did not record medication plans before admission, we assume that the higher likelihood of an already established therapy with RAS inhibitors and β-blockers at the time of STEMI may at least in part explain the lower mortality and reduced infarct size of patients with history of hypertension in our cohort. This notion is supported by the higher blood pressures recorded in these patients at time of admission, allowing for earlier and more aggressive treatment. On the same lines, previous studies investigating the effect of RAS inhibitors and β-blockers on myocardial infarction outcomes found higher prescription rates and dosing of these drugs in patients with a history of hypertension or higher systolic blood pressures on admission (20, 22). The higher prescription rates of these agents that we recorded at time of discharge in patients with a history of hypertension, however, are of proven prognostic relevance, which translates into the better short- and long-term survival in this group.

To our surprise the survival benefit of history of arterial hypertension remained persistent even in patients presenting with systolic blood pressures lower than 120 mmHg. In this patients increasing dosages of RAS inhibitors and β-blockers might not have been possible and therefore could contribute to better survival. However being diagnosed with arterial hypertension before the index event it is very likely that both medication classes would already have been administered in this patient group, allowing a primary prophylactic effect to impact survival. Furthermore, these patients profit from a lower systolic blood pressure in long-term survival as it still poses a cardiovascular risk factor.

## Effect of Long-Term Persistence

Prescription rate at discharge and persistence of medication for secondary prevention after myocardial infarction in our cohort was high. This is in line with data from other current registries (23, 24) and underlines the high standard of care in the prospective KIM registry.

A large body of evidence supports the long-term administration of RAS inhibitors in patients with left ventricular dysfunction after myocardial infarction (25, 26) and this is reflected by an IA recommendation in current guidelines (1). With reference to the HOPE and EUROPA trials, these guidelines give only a IIa recommendation for STEMI patients in general. In more recent trials, however, RAS inhibitors proved to be particularly beneficial in reducing post-infarct remodeling of the left ventricle also in unselected patients, with benefits in mortality and incident heart failure even more evident on long-term follow-up (15, 16).

On the other hand, the prognostic relevance of long-term persistence of β-blocker therapy is still a matter of ongoing debate, since no study to date has properly addressed duration of β-blocker treatment after myocardial infarction (27). Bangalore and coworkers showed in a meta-analysis that β-blockers reduced mortality in the pre-reperfusion but not later in the reperfusion era, a finding that was consistent for short (up to 30 days) as well as long term (>1 year) observation (28). In contrast, β-blocker therapy reduced mortality in a subgroup of patients with STEMI and an ejection fraction >40% from a nationwide registry (20). A most recent analysis of Danish national registry data on more than 30.000 patients hospitalized for acute myocardial infarction over the years 2003–2018 again showed no benefit of β-blocker therapy over a 3 year period (29). Hence, the higher long-term persistence on RAS inhibitors that we observed in patients with history of hypertension seems to convey a larger prognostic benefit than long-term β-blocker therapy, which was not different between our groups.

## Dosing After Acute Myocardial Infarction

While there are clear recommendations for optimal dosing of RAS inhibitors and β-blockers for the treatment of heart failure, current guidelines do not specify target doses for these agents after myocardial infarction (1). In previous registries of myocardial infarction, prescribed doses mostly ranged below 50% of target doses used in clinical trials (23, 30). Similarly, we documented average dosing for RAS inhibitors and β-blockers of 35 and 45%, respectively. Two recent studies for the first time demonstrated the relevance of target doses of RAS inhibitors on cardiovascular morbidity and mortality also for unselected patients post STEMI (22, 24). On multivariate analysis both workgroups found that higher doses of RAS inhibitors, but not β-blockers, at the time of hospital discharge were independently associated with lower long-term cardiovascular mortality and readmission for heart failure. Hence, the higher dosing of RAS inhibitors at discharge that we documented in patients with history of hypertension may have contributed to the better survival in this group.

Uncertainty remains regarding appropriate dosing of β-blockers following myocardial infarction. Data from the Korea Acute Myocardial Infarction Registry recently suggested that low dose β-blocker therapy (<25% of target) would be similarly effective as higher doses (30). Goldberger et al. even found an inverse dose-dependent effect post- myocardial infarction with the lowest mortality in patients just receiving between 12.5 and 25% of maximal β-blocker dose (23). As long as further evidence is lacking, current guidelines recommend adjusting the dose of β-blockers to limit the heart rate to 55 −60 beats per min at rest.

### Study Limitations

First, we did not assess medication plans for our patients prior to admission for STEMI treatment. Therefore, we can only speculate on the prophylactic effect of RAS inhibitors and β-blockers for the early course of the index event. However, the smaller infarct size and strong survival benefit for patients with history of hypertension that we detected within the first 24 h is an indicator for some protective effect in this group of patients. However, to comprehensively address primary prophylactic potential of statins, RAS inhibitors and β-blockers, these data are paramount to be acquired in future studies. Second, data on adherence were largely obtained relying on self-reported medication use. While this implies the inherent risk of recall bias, we are confident about the validity of our data since they are in the range of previous studies (23, 24). Third, due to missing data not the whole cohort could be analyzed for effects on mortality in the present study (Figure 1).

## Conclusion

Despite its undeniable role as independent cardiovascular risk factor, history of arterial hypertension was associated with lower short- and long-term mortality following STEMI. Patients with history of hypertension had higher systolic blood pressures at time of admission, higher prescription rates for RAS inhibitors and β-blockers at discharge as well as higher dosing and long-term persistence of RAS inhibitors. The overlapping indications for these agents in the treatment of arterial hypertension and secondary prophylaxis after STEMI likely explain the better penetration of guideline recommendations in these high-risk patients, which is of proven prognostic benefit.

## Data Availability Statement

The raw data supporting the conclusions of this article will be made available by the authors, without undue reservation.

## Ethics Statement

The studies involving human participants were reviewed and approved by Ethics Committee of the Medical Faculty of the University of Cologne. The patients/participants provided their written informed consent to participate in this study.

## Author Contributions

FH and HR: data collection, analysis, conception of study, and preparing the manuscript and editing it. PF: data analysis, figure design, and internal manuscript revision. WZ: data collection and internal manuscript revision. LU: data collection and analysis. KK: statistical analysis. CA: conception of study and manuscript editing. MH: conception of study, manuscript editing, and hypothesis formulation. All authors contributed to the article and approved the submitted version.

## Conflict of Interest

The authors declare that the research was conducted in the absence of any commercial or financial relationships that could be construed as a potential conflict of interest.

## Publisher's Note

All claims expressed in this article are solely those of the authors and do not necessarily represent those of their affiliated organizations, or those of the publisher, the editors and the reviewers. Any product that may be evaluated in this article, or claim that may be made by its manufacturer, is not guaranteed or endorsed by the publisher.

## Abbreviations

CI: Confidence interval
HR: Hazard ratio
KIM: Cologne Infarction Model
RAS: Renin-angiotensin-system
STEMI: ST-elevation myocardial infarction.
