# Supplementary material for: The Hypertension Paradox: Survival Benefit After ST-Elevation Myocardial Infarction in Patients With History of Hypertension. A Prospective Cohort- and Risk-Analysis
Source: Front Cardiovasc Med. 2022 Feb 24;9:785657. doi: 10.3389/fcvm.2022.785657 (PMC8907999; doi:10.3389/fcvm.2022.785657)
Supplement: Supplementary file 1 [file Table_1.DOCX]

**Appendices**

Tables

| All-cause | | n | Hazard ratio | 95%-CI | | *p-v*alue |
| --- | --- | --- | --- | --- | --- | --- |
| Mortality | |  |  | - | + |  |
| Total | raw | 954 | 0.37 | 0.27 | 0.51 | <0.001 |
|  | adjusted* | 644 | 0.19 | 0.10 | 0.35 | <0.001 |
| 30-day | raw | 950 | 0.41 | 0.27 | 0.62 | <0.001 |
|  | adjusted^ | 643 | 0.28 | 0.09 | 0.85 | 0.025 |
| 1-year | raw | 950 | 0.37 | 0.26 | 0.53 | <0.001 |
|  | adjusted# | 643 | 0.24 | 0.12 | 0.48 | <0.001 |
| Out-of-hospital | raw | 886 | 0.41 | 0.28 | 0.61 | <0.001 |
|  | adjusted† | 639 | 0.18 | 0.10 | 0.36 | <0.001 |

Table S1: Hazard ratio of all-cause mortality for patients with history of hypertension at different time points. Out-of-hospital mortality: mortality analysis starting at time of discharge until end of follow-up. Adjusted: linear regression analysis adjusted for age, sex, diabetes mellitus, body mass index, kidney function, Killip-class and prescription of statins at discharge. CI – confidence interval. Level of significance: *p*<0.05.

Independent predictors that affected outcome significantly:
* History of arterial hypertension, age
^ History of arterial hypertension, age, sex category, Killip-class
# History of arterial hypertension, age, sex category, kidney function, Killip-class, body-mass-index
† History of arterial hypertension, age, kidney function

| Antiplatelet/anticoagulative treatment |  | @ Discharge | | | | @ Follow Up | | | |
| --- | --- | --- | --- | --- | --- | --- | --- | --- | --- |
|  |  | Total | Hx of | No hx of | *P-*value | Total | Hx of | No hx of | *P-*value |
|  |  |  | hypertension | hypertension | hx vs. no hx of hypertension |  | hypertension | hypertension | hx vs. no hx of hypertension |
| Dual antiplatelet therapy | [%] | 93.5 | 92.9 | 93.9 |  | 23.8 | 24.5 | 19.3 |  |
| Mono antiplatelet therapy | [%] |  |  |  |  | 67.3 | 66.5 | 71.6 |  |
| Oral anticoagulans with mono or dual antiplatelet therapy | [%] | 6.4 | 7.1 | 6.0 |  | 2.5 | 2.3 | 3.4 |  |
| Oral anticoagulans | [%] |  |  |  |  | 3.7 | 4.9 | 2.3 |  |
| None | [%] |  |  |  |  | 2.8 | 2.7 | 3.4 |  |
| Overall |  |  |  |  | 0.681 |  |  |  | 0.708 |

Table S2: Relative distribution of antiplatelet and anticoagulative treatment between patients with history of hypertension and without (Hx of hypertension versus no hx of hypertension) upon discharge and follow up. Level of significance *P* < 0.05
